# Advances in the Electronics for Cyclic Voltammetry: the Case of Gas Detection by Using Microfabricated Electrodes

**DOI:** 10.3389/fchem.2018.00327

**Published:** 2018-08-10

**Authors:** Giorgio Pennazza, Marco Santonico, Luca Vollero, Alessandro Zompanti, Anna Sabatini, Nandeesh Kumar, Ivan Pini, William F. Quiros Solano, Lina Sarro, Arnaldo D'Amico

**Affiliations:** ^1^Unit of Electronics for Sensor Systems, Department of Engineering, Campus Bio-Medico University of Rome, Rome, Italy; ^2^Unit of Computational Systems and Bioinformatics, Department of Engineering, Campus Bio-Medico University of Rome, Rome, Italy; ^3^Department of Electronic Engineering, University of Rome Tor Vergata, Rome, Italy; ^4^LIntes Research Laboratory, Teramo, Italy; ^5^Laboratory of Electronic Materials, Devices and Components, DIMES, Delft University of Technology, Delft, Netherlands

**Keywords:** voltammetry, lock-in, gas sensors, electronic interface, multivariate analysis, multivariate pattern analysis, oxygen, carbon dioxide

## Abstract

This paper presents an advanced voltammetric system to be used as electronic tongue for liquid and gas analysis. It has been designed to be more flexible and accurate with respect to other existing and similar systems. It features improved electronics and additional operative conditions. Among others these include the possibility to optically excite the solution and to treat the output signal by a differentiation process in order to better evidence the existence of small details in the response curve. Finally by the same type of tongue preliminary results are shown dealing with O_2_ and CO_2_ concentration measurements in appropriate solutions.

## Introduction

Cyclic voltammetry (CV) is one of the most-important and most-widespread applied electrochemical techniques (Legin et al., 1997; Tahara and Toko, 2013). This technique has been also utilized in the design and development of the so-called Electronic Tongue (ET), as multisensory system for liquids.

An ET can be imagined as an ensemble of chemical sensors able to mainly operate in liquids and give chemical images of them. As we know the different tastes that humans can detect by the tongue are five in number and are called as follows: saltines, bitternes, sweetnes, sournes, and umami; for each of them about 100 receptors are available in humans for redundancy purpose.

The ET has been employed to discriminate mainly chemical species in liquids (Legin et al., 1995, 1997, 1999a; Vlasov et al., 2005) by an array of chemically sensitive devices such as ion-selective electrodes characterized by different specificity and through a typical statistical analysis such as Principal Component Analysis(PCA) and Partial least Square(PLS) or specific neural networks (Krantz-Rülcker et al., 2001; Legin et al., 2004; Rudnitskaya and Legin, 2008). In this case the total specificity (one sensor for one chemical specie) for each sensor is proved to be not necessary at all. As a matter of fact, in practice it happens that most of the employed sensors for the matrix are characterized by a rater low specificity(each sensor sensitive to more than one chemical specie). On the other hand it is possible to develop specific taste sensors (biosensors) (Rudnitskaya et al., 2008) that only respond to a specific taste type (Vlasov et al., 2000). It worth pointing out that also for the human tongue, although characterized by sufficiently well localized taste bio-receptors, they seem to be non-perfectly specific.

The (ET) looks like an electronic nose (Legin et al., 1999b; Winquist et al., 2000; Santonico et al., 2013) whose typical ambient is gaseous in character but at the end also in this case a compound image is obtained.

Although many papers have been written so far on ET, technical aspects that are dealing with electronic interface performance, especially with respect to accuracy and versatility have not sufficiently been addressed. For this reason we have oriented the content of this paper toward those technical aspects that are dealing with electronic interface performance when the accuracy and versatility are among the most important parameters to take into account.

Essentially three are the main detection techniques employed for the liquid investigation when an ET is used: (a) Matrix ion selective electrodes, (b) Amperometric analysis, and (c) Voltammetric analysis.

Referring to point (a) we have to consider the use of multi-metal head as an ions multi-sensors system where chemical potentials are measured and associated to the chemical species in solution (Rudnitskaya et al., 2008). In this case techniques such as the PCA-PLS can be advantageously used for the determination of the chemical species present in solution.

Point (b) refers to a technique where red-ox reactions are put in evidence by the measure of the current once a suitable voltage is applied to the electrodes.

Among the different possibilities to deal with this problem, we will confine our attention to the last one, and illustrate the new voltammetric system.

In particular, we will consider along this paper aspects dealing with the improved (ET) related to:

- small signal detection by very highly selective filters;
- low noise amplifiers;
- optical systems for the injection of light power in the proximity of the working electrode;
- high input dynamic range(IDR);
- improved input flexibility including DC bias and ramp together;
- possibility to zoom inside the IDR;
- gas detection in suitable liquids (example with O_2_ and CO_2_) (Pennazza et al., 2017; Santonico et al., 2017);
- technology for miniaturized electrodes;
- differentiation of the output signal for higher resolution analysis;

Moreover, In this paper we illustrate some advanced features included in the designed system and comment on the versatility of the information that can be gained by its applications. The possibility of measuring different types of gas (O_2_ and CO_2_) fluxed through liquids is also shown.

## Materials and methods

In this section the designing and development of the last sensor system here used is presented. The description starts by the introduction to the sensing system, passing through the innovative device realized by an advanced cyclic voltammetric structure. The interface developed for liquid analysis is illustrated and the sub-section devoted to the electrodes and to the calibration procedure closes the experimental section.

### The sensing system

The sensing system is based on an electrochemical analytical technique called cyclic-voltammetry, usually used to study a compound, a biological material, or an electrode surface. In voltammetry a time-dependent potential is applied to an electrochemical cell and the resulting current is measured as a function of this potential.

**Figure 1:**
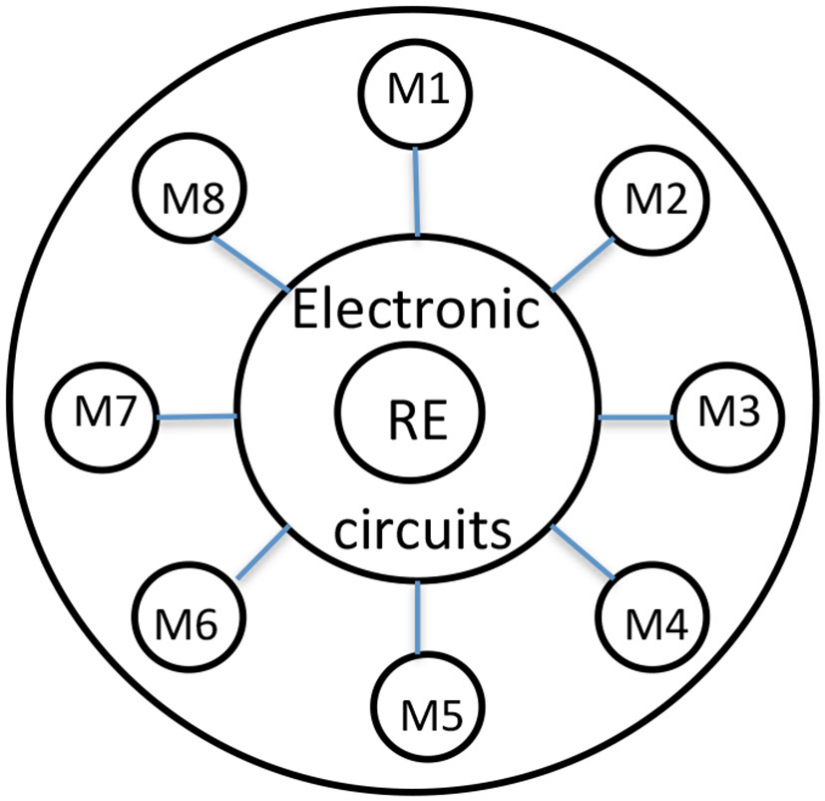
Shows a schematic of a typical ion selective head. Schematic representation of the electronic tongue head made by 8 different metals as ion sensors. The reference electrode is in the center. The electronic part is located at the back.

The electrochemical cell used in voltammetry is, in most cases, made of three electrodes, respectively called **working (WE)**, **reference (RE)**, and **counter (CE)** electrode (Figure 1). A time-dependent potential excitation signal is applied between the working electrode and the reference electrode and the current that flows between the working and auxiliary electrodes is measured. Current peaks, observed at specific applied voltages, are due to specific redox reactions running on the working electrode surface. Cyclic Voltammetry (CV) consists of cycling the potential of the reference electrode. The sensing system essentially consists of the following elements: electrochemical cell, electronic interface, microcontroller unit, PC for data analysis.

**Figure 2:**
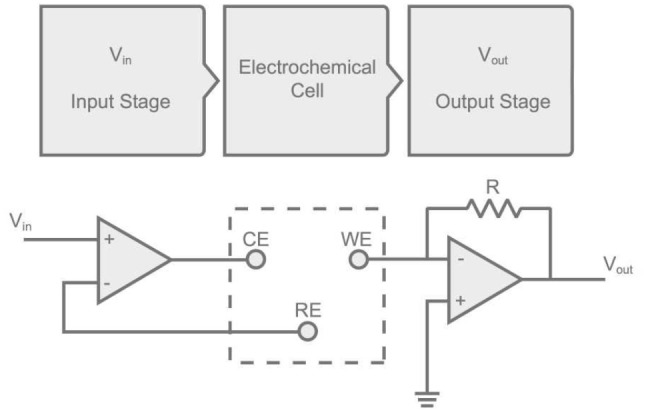
Scheme of the signal conditioning circuit stage.

The signal conditioning stage applies the time-dependent potential between the working and reference electrodes and converts the current flowing between the working and the auxiliary electrodes into a voltage signal, then digitally acquired by the ADC conversion stage (Figure 2).

### The advanced cyclic voltammetric structure

**Figure 3:**
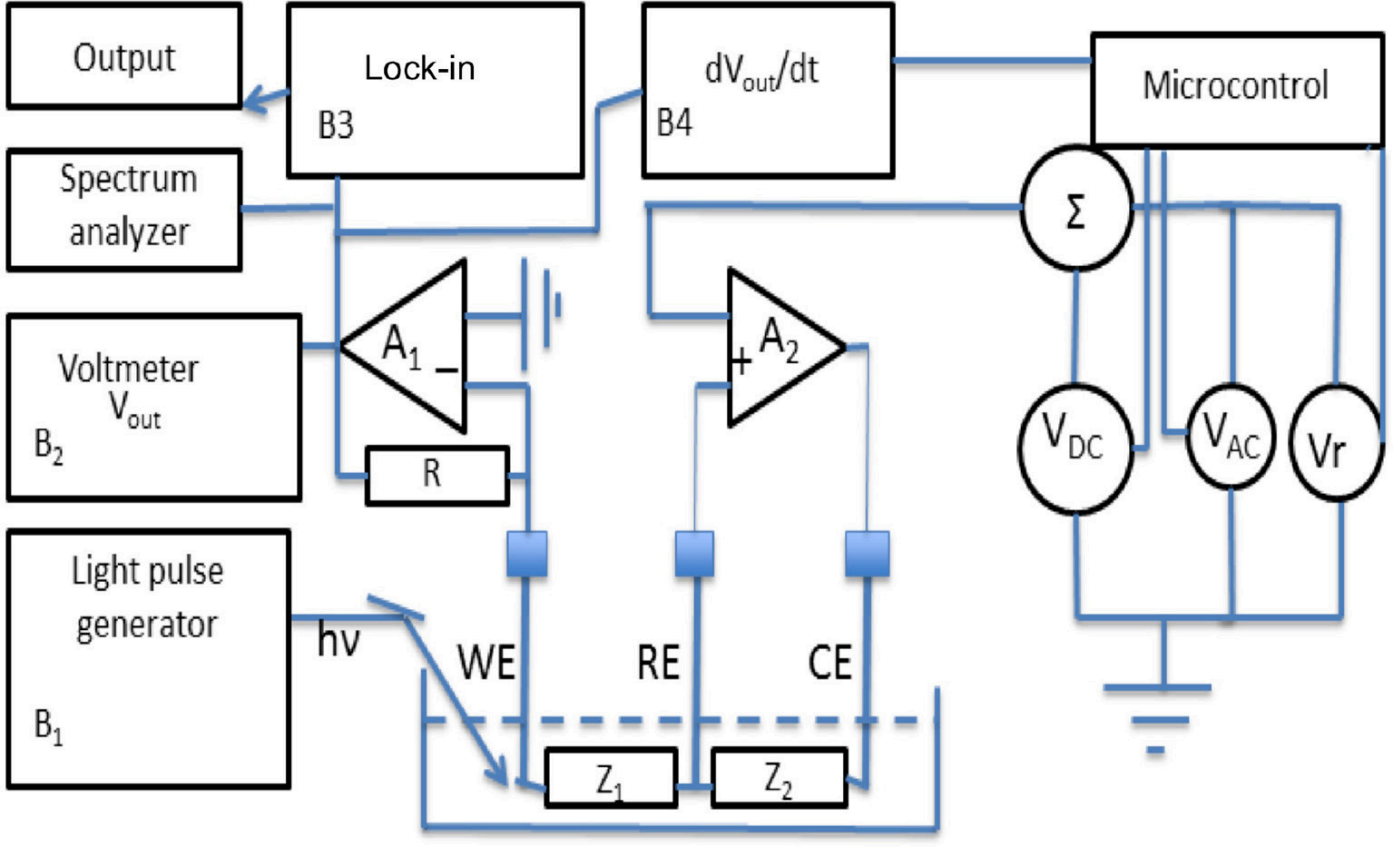
The advanced voltammetric structure described in this paper.

Figure 3 represents an advanced and rather complete voltammetric electronic system characterized by high resolution, operating with three conventional electrodes (WE), (CE), and (RE).

V_DC_ represents a bias voltage, V_AC_ represents a very small amplitude sinusoidal voltage which is added to V_DC_ and V_r_ represents both the positive and negative time dependent ramp function. These three types of signals can be applied one at the time or coupled to each other in order to give rise to different analysis methodologies. Finally a lock-in amplifier is used to measure the output of the trans-impedance low noise amplifier (A_1_) The low noise amplifier A_2_ has the duty to inject current into the solution by the (CW) and to apply, as an example, both a V_DC_ voltage to the reference electrode and the ramp voltage V_r_.

The voltage ramp is designed to have a variable slew rate from 0.1 up to 10 mV/s. The system can be programmed through the microcontroller to infer a zoom action in particular points of interest, determined by a significant polarization value of the (RE).

Another important strategy that can be adopted in the system represented in Figure 3 is the presence of an electronic block (B4) able to perform the first time derivative of the output voltage. This is proved to be useful in those cases (complex solutions) where varieties of red-ox reactions can take place, in order to better evidence hidden details of the current changes. Block B2 does represent the output of the trans-impedance amplifier A1, while B1 represents the generator of suitable light pulses.

A further strategy considers the output voltage divided in many samples (from 500 up to 1,000) according to the selected division in the time domain. This strategy allows the transformation of an (ET) in a huge number of equivalent sensors with the advantage associated to the matrixes methodology.

### Electronic tongue for liquid analysis: interfaces

It has been demonstrated that the ET is a practical tool for the investigation of red-ox reaction phenomena in different kind of liquids.

Here we will enter some details aiming at exploring the intrinsic mechanisms of the varieties of electronic circuit behavior in real operative conditions.

Furthermore, we will comment on the main results obtained so far by this technique, indicating the possible technical improvements that are necessary to achieve better results in terms of sensitivity and resolution.

Any time that the design involves processing lines for small signals analysis, made by amplification steps, filtering, conversions, all embedded in noise, it is of up most importance a careful characterization of the source of signals in terms of output current or voltage.

This is normally done estimating the impedance value, defining its structure (differential with or without common mode voltages or currents or unbalanced with respect to a reference point, etc.) and the signal to noise (S/N) ratio. This would allow to face the most convenient measurement strategy. In those cases where the S/N is greater than one, usual techniques of amplification and filtering can be employed taking care that the small S/N perturbation should be the main concern. In those case where the S/N ratio is less than one it is necessary to consider particular techniques listed below and easy to find in the technical notes:

1. Lock-in amplifiers (analog or digital) with only one reference frequency.
2. Waveform averagers such as for instance the “box car integrators.”
3. Autocorrelators and crosscorrelators.

In this paper only the lock-in amplifier operating with a single reference signal is taken into a brief consideration, because it is the most utilized in the sensor context especially when the noise introduces measurement problems.

The Lock-in amplifier can be seen as a very narrow filter whose central frequency can be selected according to the operating frequency of the experiment under test.

It is basically a phase sensitive ac frequency selective voltmeter that compares the input signal against a reference signal in a phase sensitive circuit, producing a dc level proportional to the part of the input signal which is synchronous with the input and in phase with the reference.

**Figure 4:**
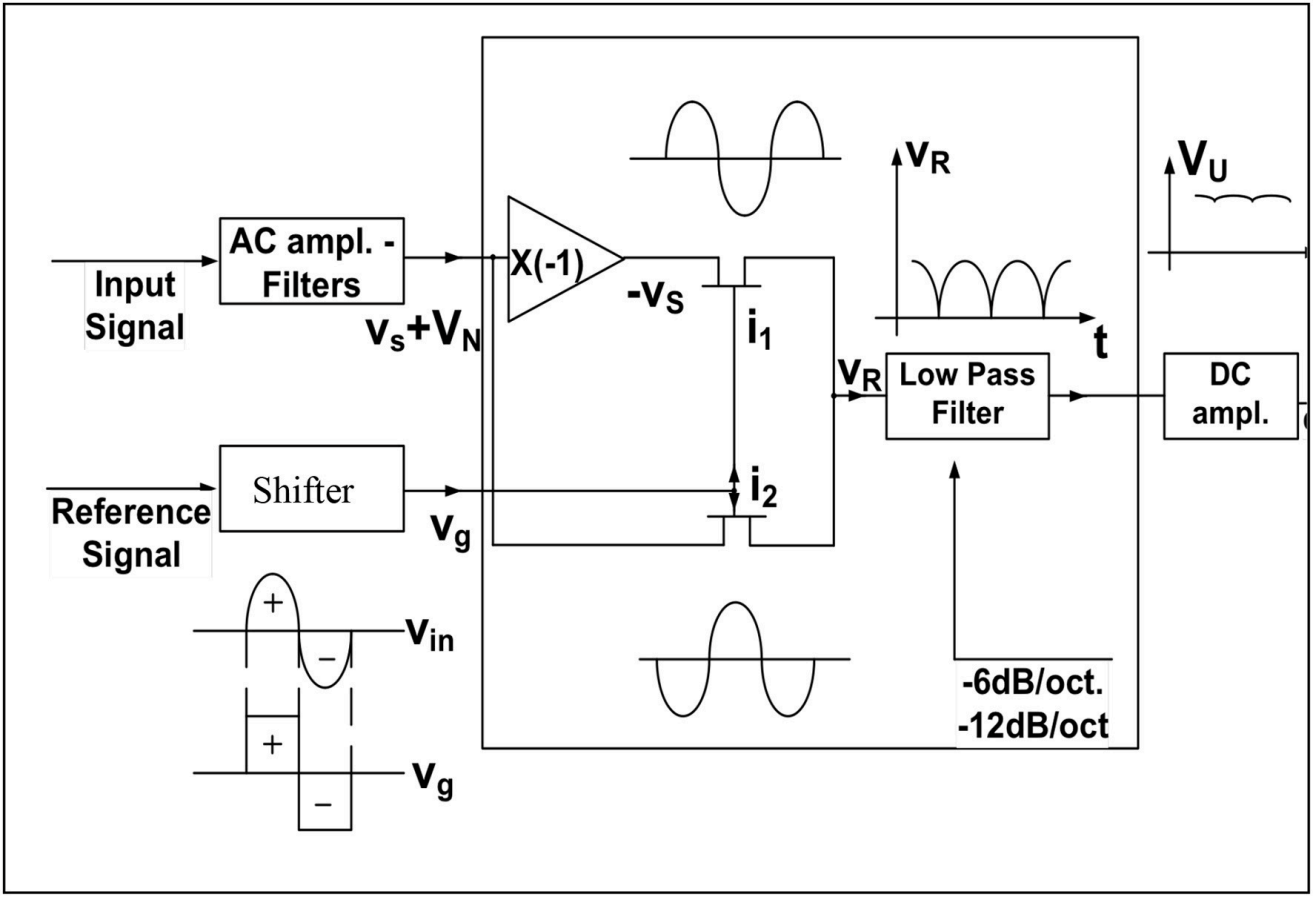
Schematic of a lock-in amplifier.

The basic principle of a lock-in amplifier is shown in the following Figure 4.

The reference signal is the input of a phase shifter which has the duty to form two output signals able to drive the two gates I_1_ and I_2_ in order to get the full rectification of the sinusoidal input signal, as shown in the node V_R_. Of course the noise level can be well above this rectified signal. Only after the low pass filter action the noise will be reduced leaving the average output value with a signal to noise ratio larger than one as expected.

**Figure 5:**
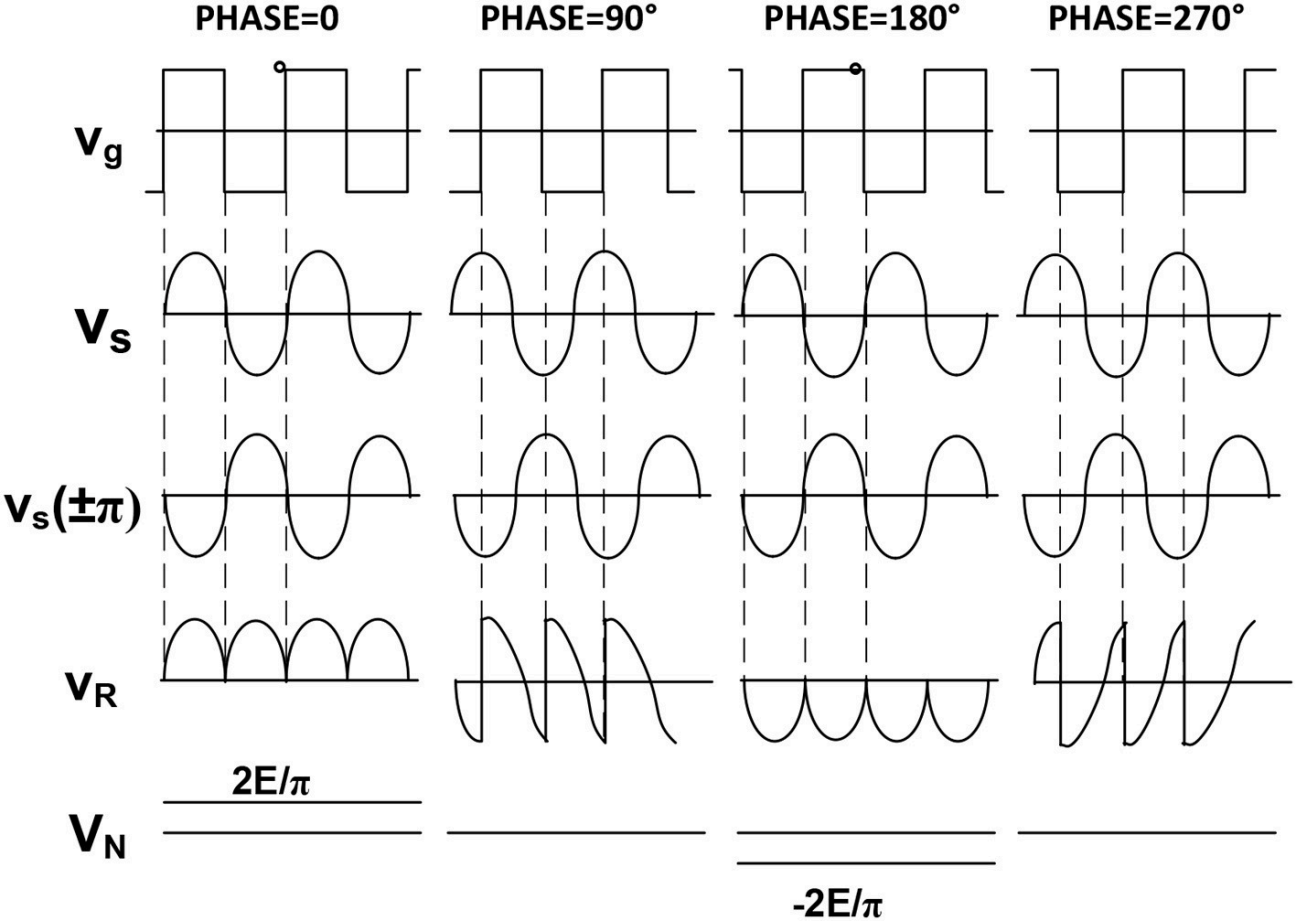
Phase implication on the detection procedure. The output can be positive or negative according to the phase difference between signal and reference. Two are the input signals: V_i_ of which it is desirable to measure the amplitude, and V_r_ which represents the reference signal.

Figure 5 shows the paramount importance of having a phase shifter in the reference block. In fact, only if this phase between the input signal and the reference one is 0° or 270° we have a sound meaning of the DC output which reaches the maximum amplitude.

The output V_o_ of the synchronous detector is given by the following product:

$$\begin{array}{l} {\text{V}}_{\text{R}}={\text{V}}_{\text{i}}*{\text{V}}_{\text{g}} \end{array}$$

where V_i_ = V_s_ + V_n_

The low pass filter located at the output of the synchronous detector will reduce the noise leaving a DC component representing the average value of the rectified sinusoid.

(1) $$\begin{array}{l} V_{OUTPUT}=\frac{1}{T}\cdot \overset{T}{\underset{0}{\int }}\left(V_{N}+V_{S}\right)dt=\frac{1}{T}\cdot \overset{T}{\underset{0}{\int }}V_{S}dt+residual.noise \end{array}$$

where *T* is the averaging time, i.e., the time necessary to perform the measurement and to reduce the noise.

The higher T the most effective the noise reduction is.

So a longer waiting time results in a more accurate measurement.

The S/N ratio improvement achievable through the use of a lock-in amplifier can be expressed by the ratio of the signal to noise ratio at the output to the signal to noise ratio at the input.

This improvement can also be expressed as the square root of the signal source bandwidth divided by the equivalent noise bandwidth of the lock-in amplifier, which is given by 1/4τ (τ = *RC*) in the case of 6 db/octave output filter.

So in order to increase the improvement ratio by a factor of 10, it is necessary to increase the output filter time constant by a factor 100.

It is worth mentioning that a lock-in amplifier can be seen as a narrow filter whose Q (quality factor of the filter) can be expressed as $Q=\frac{f_{0}}{\Delta f}$ where *f*_0_ is the operating frequency and Δ*f* is the bandwidth of the output filter given by $\Delta f=\frac{1}{4\tau }$ and centered in the origin of the frequency axes.

The higher the operating frequency is, the higher both the Q and the noise rejection are.

**Figure 6:**
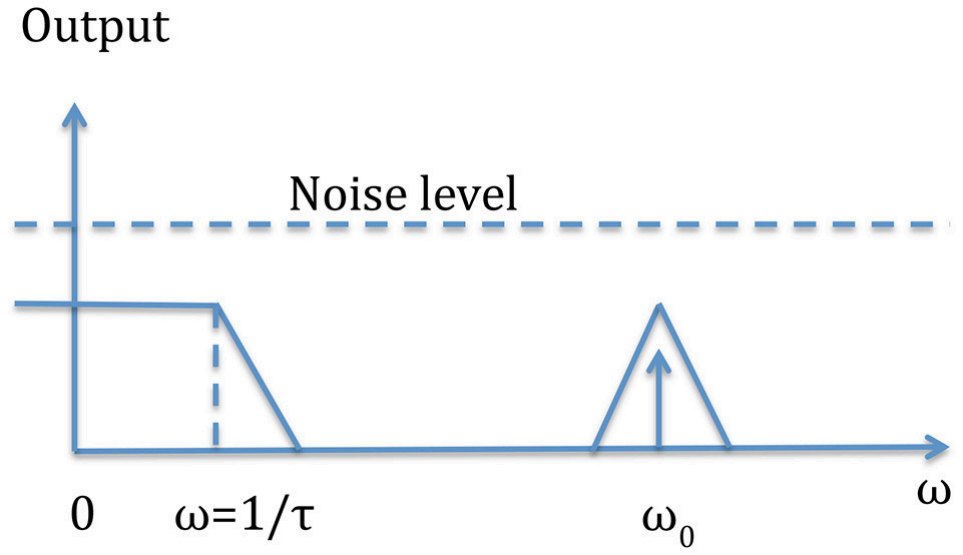
This figure shows the intrinsic mechanism of the output filter. The input signal of frequency f_0_ = ω_0_/2π, embedded in noise is translated to a DC signal with a noise only determined by the low pass filter near the frequency zero.

In some applications the signal is modulated at a given frequency so integrated lock-in amplifiers can be advantageously used to achieve a better S/N ratio. Figure 6 schematically shows how the lock-in filter works: the equivalent filter is that of a low pass filter whose width can be reduced increasing the time constant. So in order to have a high Q filter we have to increase the measuring time. This means that a trade-off must be reached between a low signal to noise ratio and the measurement time.

The system has been tasted from the noise point of view, especially as far as the trans-amplifier (A1) is concerned. It has been designed and constructed by assembling discrete bipolar transistors. The measured noise spectral density in the bandwidth ranging from 1 Hz up to 100 KHz has been as low as 1 nV/(Hz)^1/2^. This has permitted to reach high resolution values during the measurements.

### Electrodes

**Figure 7:**
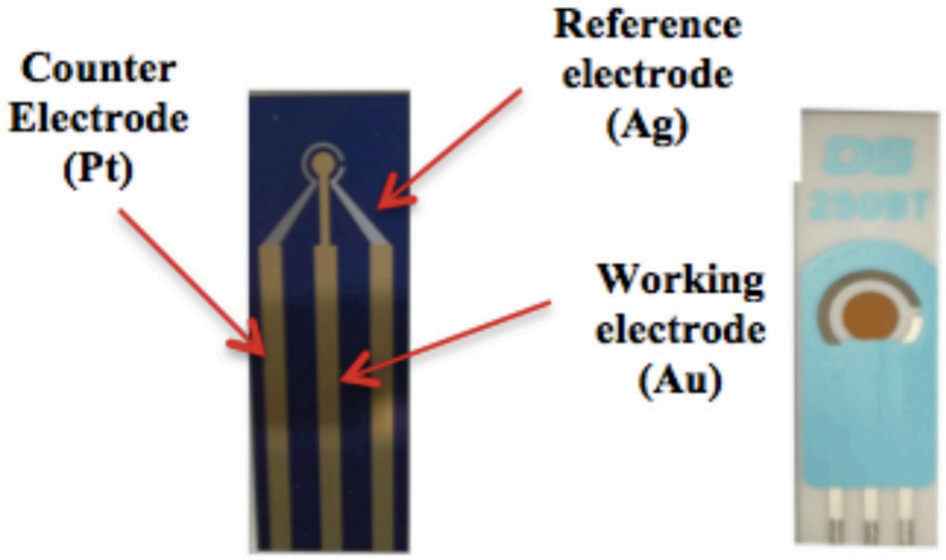
Micro-fabricated electrode (left) and Commercial screen-printed electrode (right).

The sensing system was tested with two types of electrodes (reported in Figure 7):

- A Micro-fabricated electrode,
- A commercial screen-printed electrode.

#### Commercial screen-printed electrodes (SPE)

The system can use commercial screen printed electrodes made by Dropsens (ref. code 250BT). The working electrode is made of gold and has a diameter of 4 mm, the counter electrode is made of platinum, the reference electrode is made of silver and all the other electric contacts are made of silver.

The supporting material is ceramic and the total thickness of the electrode is 1 mm. The thickness of the metal material screen-printed on the electrode is about 1 μm. The surface roughness is quite high.

#### Micro-fabricated electrodes (MFE)

Micro-fabricated electrodes were designed to improve the performances of the sensing system. The main aim was to have a more effective control on the fabrication of the electrodes. In fact, using a micro-fabrication process it is possible to control the thickness of each metal layer, the purity of the materials and the surface roughness.

The electrodes were fabricated in the Else Kooi Laboratory of the Delft University of Technology.

The metals used in the micro-fabricated electrode are the same as those of the SPE. Two batches of the MFE were fabricated: one with a 4 mm-long working electrode (in practice with geometry and size identical to the SPE type); another with the same geometry of the SPE, but with a smaller working electrode diameter, namely 1 mm. In the following we will refer to the first and to the second respectively as MFE-4 and MFE-1.

**Figure 8:**
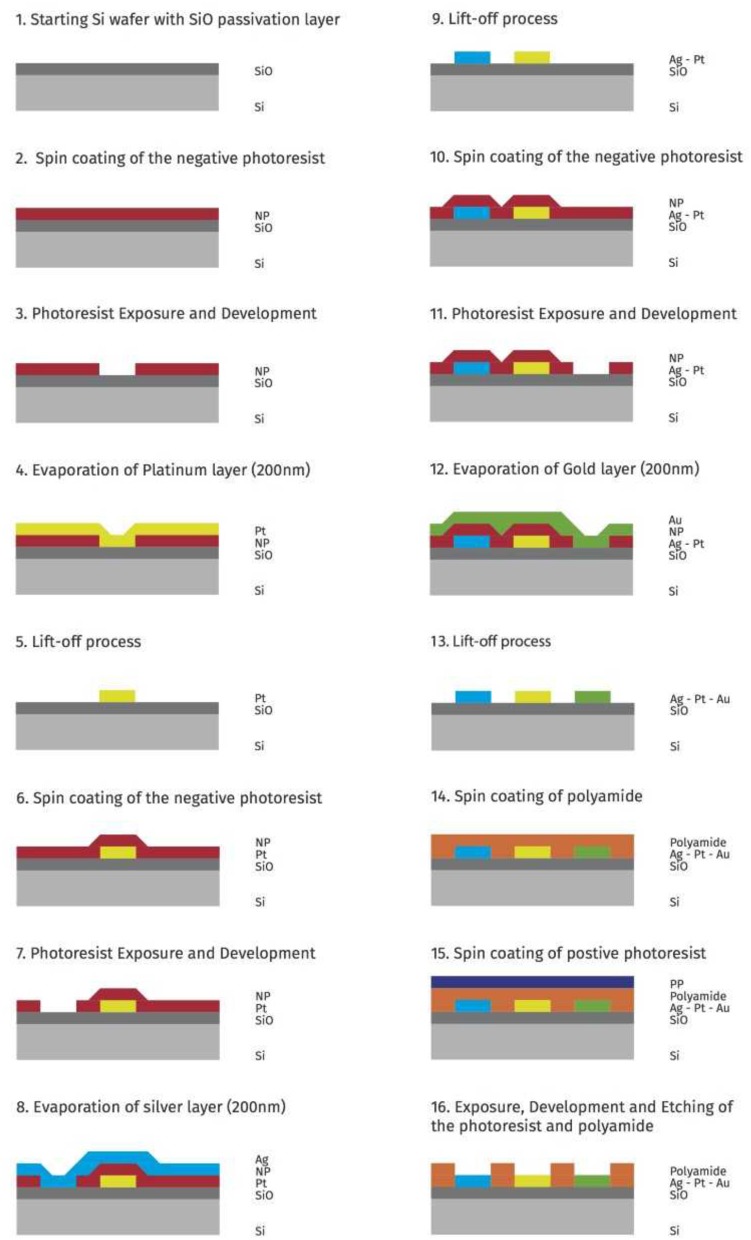
Main fabrication steps for the microfabricated electrodes.

The fabrication process is schematically illustrated in Figure 8. The starting substrate is a 4” diameter silicon wafer on which 300 nm of thermal oxide for substrate isolation is grown (Figure 8.1). The first metal electrode, the counter electrode, is formed using a lift-off process (Figures 8.2–8.5). First, a 1.5 μm-thick photoresist layer is spun and pre-baked at 100°C for 60 s. The electrode pattern is then exposed using contact exposure and developed using MF-300 series developer. After development, a deep UV post-exposure and an oxygen plasma flash to clean are performed to condition the photoresist for the lift-off process and to remove any residue from the surface where the metal is to be deposited, respectively. Subsequently, a 200 nm-thick platinum layer is deposited by thermal evaporation. The lift-off (removal of the photoresist and the undesired platinum) is done in an ultrasonic bath of NMP (N-Methyl-2-pyrrolidone) at 70°C. The substrate is rinsed in DI water and dry with a spin dryer to guarantee a clean surface prior the following second and third metal electrodes deposition and patterning.

To fabricate the reference electrode (Ag) and working electrode (Au), a 200 nm-thick layer is evaporated for both metals. The patterning and lift-off process is performed as previously described (Figures 8.6–8.13).

Finally a 5 μm polyimide layer is deposited by spin coating (Figure 8.14) and patterned to expose the contact areas to the solution and to isolate the metal lines. The polyimide is patterned (Figures 8.15,8.16) using conventional lithography. The unwanted polyimide layer is etched in 25% TMAH (Tetramethylammonium hydroxide) solution. Finally, the masking photoresist is stripped in oxygen plasma and the polyimide is thermally cross-linked in a vacuum oven at 350°C more details can be found in the Supplementary Material.

### Calibration procedure

In order to compare different behaviors of the electrodes described before, a dedicated electronic interface has been designed, based on a comparator coupled with a trans-impedance circuit. The system provides signals to an ADC device and the acquisition board is based on an ATMEL 328 microprocessor with a 12 bit converter. The input signals are generated by an internal function generator which can provide different periodic waves (square, triangular, sine) at different frequencies.

The sensor system has been calibrated via a specific sampling procedure. In order to reduce the effects of sampling errors an apparatus composed of a mass flow controller (MFC) with a minimum dilution of 1:400 was used.

**Figure 9:**
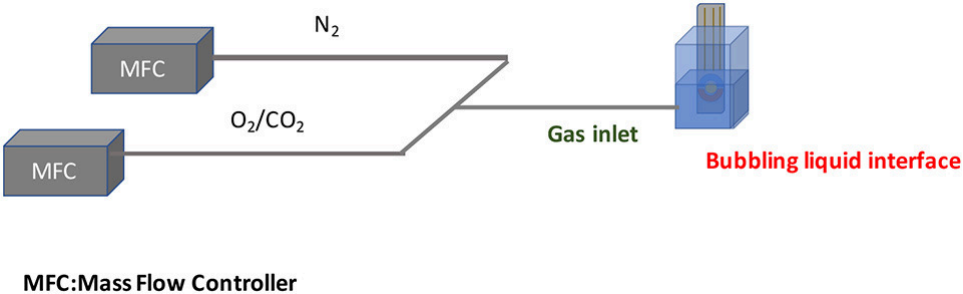
Schematic overview of the sampling protocol.

In Figure 9 a schematic drawing of the experimental set-up is shown.

The *MFC* was used in the sensor calibration to provide different concentrations of gases (in this work CO_2_ and O_2_). The output of each MFC device is mixed in order to obtain different percentage of N_2_ as carrier gas and of the target gas (O_2_, CO_2_). Both commercial electrodes and custom micro fabricated electrodes described before were immersed in a target solution of 4 ml in volume. The solution has to be bubbled with a one of the gas target for 30s. The high-resolution of the system allowed the analysis of even small charge changes in the solution (Pennazza et al., 2017).

In particular the tested solutions are: (1) physiological solution; (2) a mixture of a suitable physiological solution in distilled water with 1:20 ratio. This ratio has been experimentally determined in order to give a sufficient conductivity to the solution.

When the electrodes are immersed into the solution, the electronic interface activates the electrodes by applying one of these three wave forms: triangular, sine and square.

The gas targets used for the calibration (O_2_ and CO_2_) have been considered with concentration levels in the range of [0–100%].

## Results: preliminary tests on SPE, MFE4 and MFE1

A preliminary set of tests was performed by the different electrodes in order to verify their affordability and the correct functionality of the electrochemical working principle. To this purpose, the electrodes were tested using a high-end potentiostat by Metrohm, the Autolab PGSTAT302N. SPE and MFE4 were compared by using a solution of DI water containing concentrations (0.1, 0.5, 1, 2, 3, 4 mM) of ferrocyanide ions Fe(CN)4-. This kind of ion was chosen because it produces a reversible redox reaction and it is widely used in electrochemical experiments. The experiments were done applying a triangular wave ranging from −0.25 and 0.6 V with a scan rate of 0.04 V/s to the reference electrode.

The output current data have been interpolated by a quadratic function and the sensor characteristics have been evaluated as shown below in Table 1.

**Table 1:** Comparison of the two electrode models.

	**Screen-printed electrodes (SPE)**
Interpolating function	2.93·10^−6^*x*2+3.28·10^−6^*x*+2.36·10^−6^
R-square factor	0.9839
Sensitivity function	5.86·10^−6^*x*+3.278·10^−6^
LOD (noise current 10 nA)	0.0031 *M*
	**Micro-fabricated electrodes (MFE)**
Interpolating function	9.51·10^−7^*x*2+1.13·10^−5^*x*+3.54·10^−6^
R-square factor	0.9939
Sensitivity function	1.9·10^−5^*x*+1.13·10^−5^
LOD (noise current 10 nA)	0.0009 *M*

**Figure 10:**
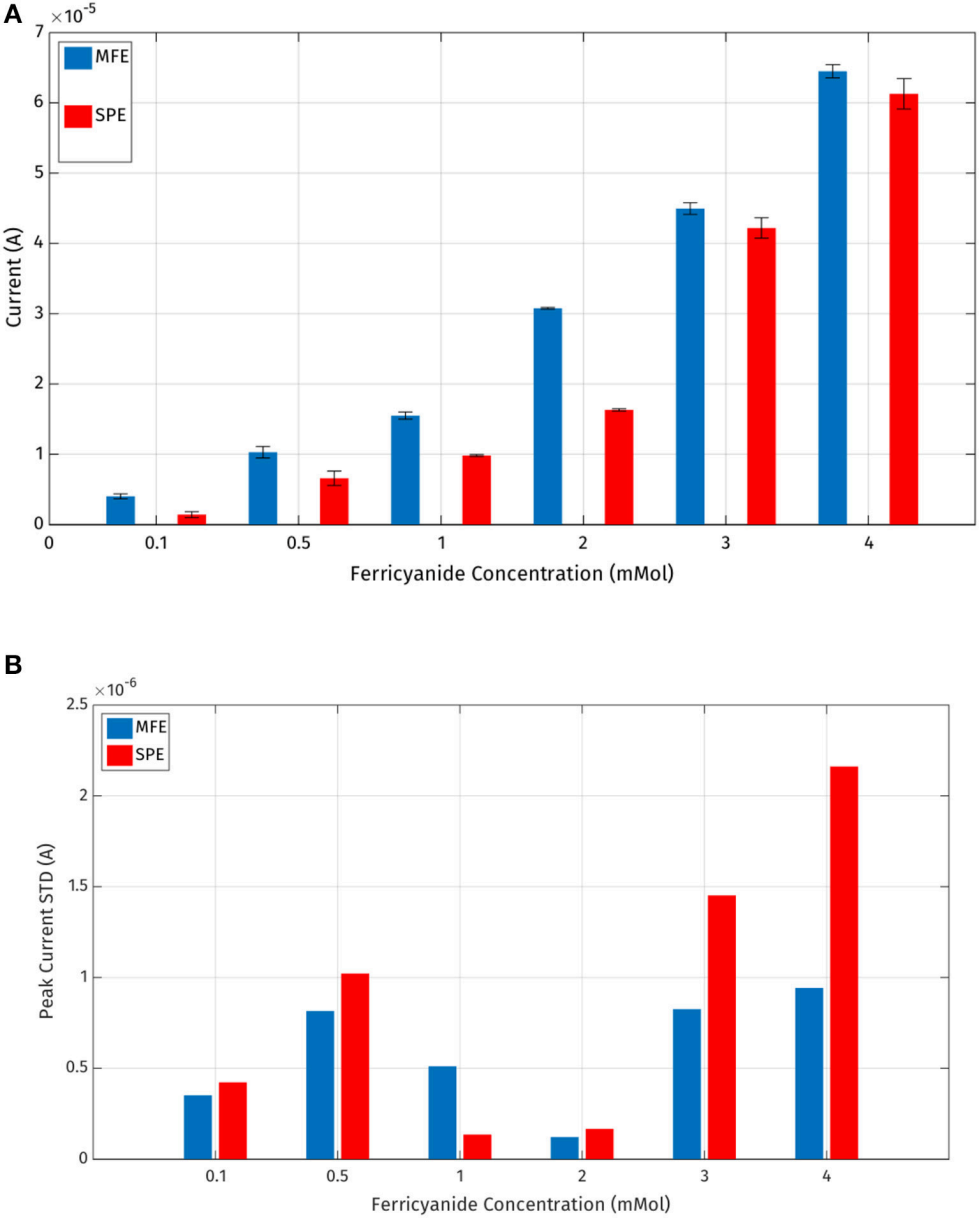
**(A)** Mean values and standard deviations of the output current peaks registered by the MFE and SPE reported in Table 1; **(B)** standard deviations of the output current peaks.

It is also possible to evaluate the precision of the experimental data generated with the two types of electrodes. Considering Figures 10A,B, it is possible to compare standard deviations of five repeated measurements per concentration for each electrode: it's evident that micro-fabricated electrodes produce more precise measurements with a lower standard deviation error and a higher current peak mean value.

Thus, it has been demonstrated experimentally that the micro-fabricated electrodes should allow a slightly lower LOD (Limit of Detection) also producing more repeatable measurements.

At this point the effectiveness of MFEs was demonstrated. Thus, a further cyclic voltammetry experiment was conducted using a solution of DI water containing a concentration of 4 mM of ferrocyanide ions Fe(CN)4-, in order to verify MFE1 applicability. The principal electrical and fabrication parameters calculated for SPE, MFE1, and MFE4 are reported in Table 2.

**Table 2:** SPE vs. MFE1 as CO_2_ and O_2_ sensors.

	**Commercial electrode**	**Custom electrode**	**Custom electrode with reduced size**
*S_*Pt*_ exposed* [mm^2^]	2.67	2.67	0.67
*S_*Ag*_ exposed* [mm^2^]	0.83	0.83	0.21
*S_*Au*_ exposed* [mm^2^]	12.47	12.47	3.12
*R_*Pt*_ [Ω]*	1.7		11.35
*R_*Ag*_ [Ω]*	0.4		1.85
*R_*Au*_ [Ω]*	0.6		1.71
*Peak Current [uA]*	About 60	About 60	About 6

Gas sensor calibration has been performed in two steps, only using MFE-1 because the main interest related to this technological step was size reduction. In the first step a regression model has been built for both electrodes. The measurement sequence was randomized in order to avoid memory effects.

The best results were evaluated considering the root mean square error in cross validation (RMSECV) relative to different responses of sensors to specific wave form signal inputs. A RMSECV of about 10% for MFE1 and a RMSECV 9.65% for SPE were obtained.

**Figure 11:**
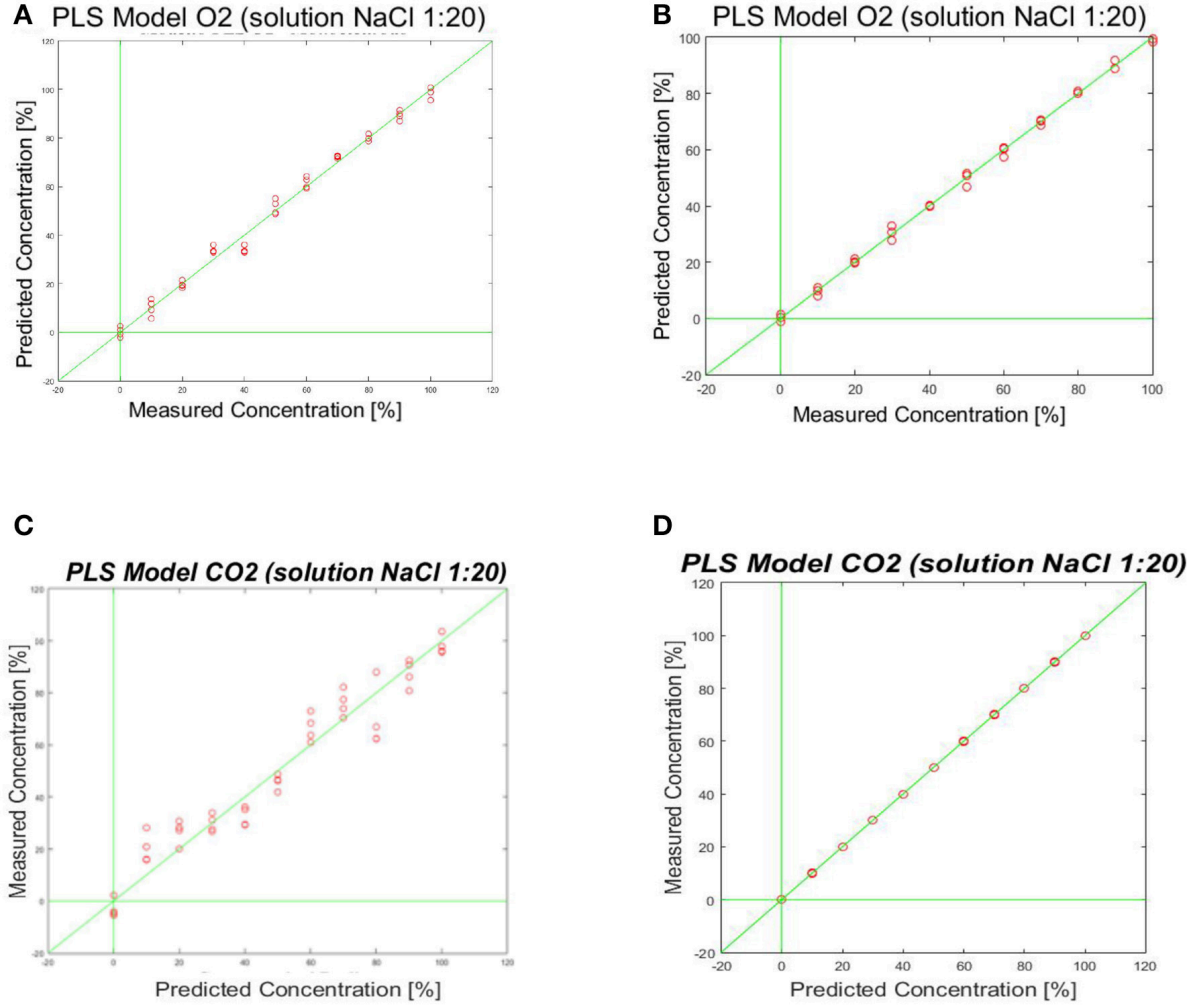
Shows the prediction ability of the calculated PLS-Model. **(A,B)** PLS-DA model obtained by fingerprinting of SPE (Figure 7 right) and MFE1 (Figure 7 left); **(C,D)** PLS-DA model obtained by fingerprinting of SPE (Figure 7 right) and MFE1 (Figure 7 left).

For the CO_2_ sensor, the best results were obtained using a sine wave form as input signal. The same procedure has been adopted for O_2_ sensor. In Figure 11 the prediction ability of the calculated PLS-DA model is represented.

Also in this case the best results were evaluated considering the root mean square error in cross validation (RMSECV) relative to different responses of sensors to specific wave form signal inputs. A RMSECV of about 10% for MFE1 and a RMSECV 21.3% for SPE were obtained.

**Figure 12:**
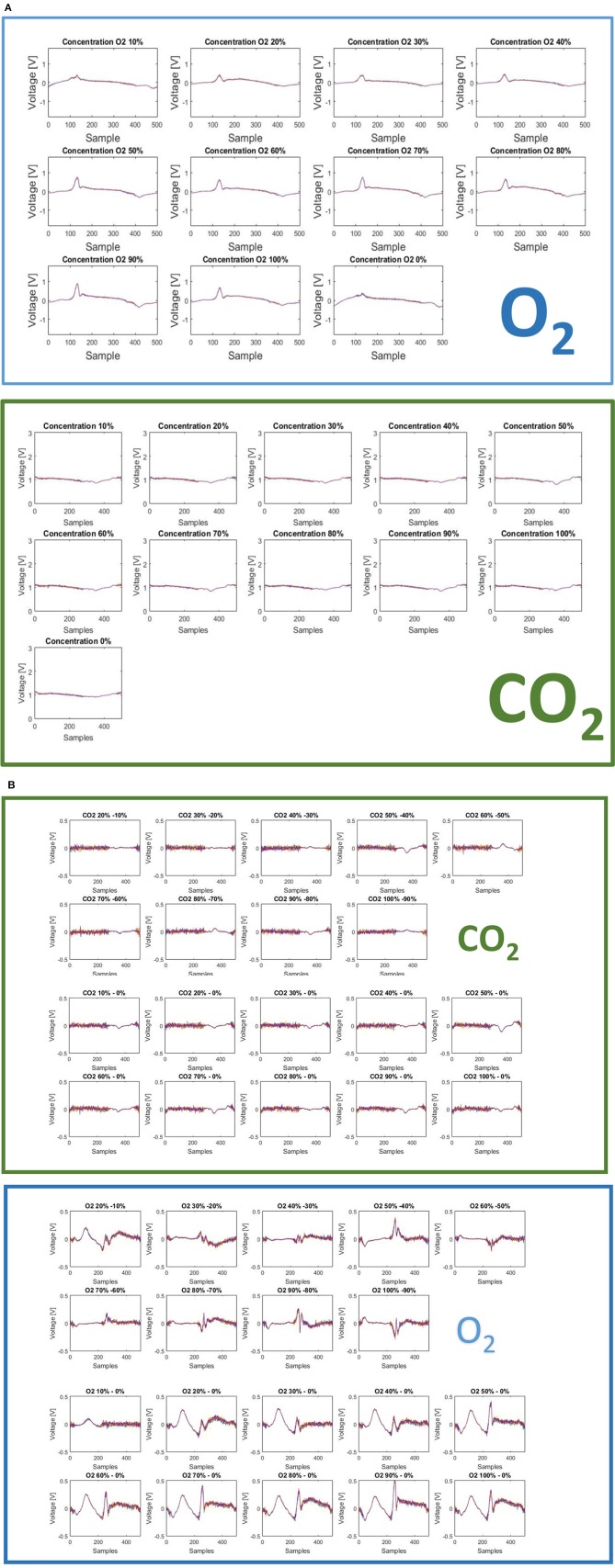
**(A)** The fingerprints here presented show two important features of the sensor: (1) measurable differences are present for different concentration level of CO_2_ and different concentration levels of O_2_; (2) measurable differences are indeed present for different gases: CO_2_ and O_2._ These two aspects account for a useful intra-gas and inter-gas fingerprint variability, which permits gas identification and calibration. **(B)** Difference of finger printings among different responses to gas concentration [CO_2_ green panel (above), O_2_ blue panel (below)].

All the measurements have been executed in triplicate for each concentration. The best performance of MFE1 has been obtained by analyzing the multidimensional fingerprinting outputs. In Figures 12A,B the fingerprints for each concentration of O_2_ and CO_2_ are reported.

Two different finger printings can be easily observed for the two gases. This aspect is interesting because, on this basis, it is possible to characterize a specific gas elaborating the multidimensional informative content provided by each fingerprinting. Another advantage could come by adopting a feature extraction strategy of on the raw data. In particular, the system provides 500 current values corresponding to the interaction of the sensor with the solution modified with gas. By selecting a reduced number of features (less than 500), which is useful for a specific target application, it is possible to optimize the measurement time, thus reducing the exposition of the electrode to the gas. This last point could also improve sensor repeatability, reproducibility and life time.

It is worth comparing the performance of this prototype gas sensor with the commercial solution currently available. This comparison is well illustrated in Table 3.

**Table 3:** Comparison table of the proposed gas sensor with some reference standard at the state of the art.

	**TR250Z Oxygen sensor**	**SST LuminOx**	**LFO2-A2 Oxygen sensor**	**Device presented in this work**
Technology	Zirconium dioxide (ZrO_2_)	Fluorescence–based optical technology	Electrochemical	Electrochemical
Dimensions	89 × 70 × 13 mm	20 × 20 × 12.5 mm	20 × 20 × 17.4 mm	40 x 40 x 18 mm
LOD	0.01% O_2_	0.01% O_2_	0.1% Oxygen	round 10% for both O_2_ and CO_2_
CO_2_ and O_2_ sensitivity	Oxygen	Oxygen	Oxygen	BOTH
Power consumption	24 VDC	4.5–5.5 VDC	0.6 VDC	±1.8 VDC

From this table it is evident that the proposed solution is competitive in terms of dimensions and power consumption, and it is interesting its ability of measuring both oxygen and carbon dioxide. Regarding the Limit of Detection, the results obtained must be improved.

## Conclusions

In this work we have described a more complete voltammetric system called electronic tongue for the determination of the features related to red-ox reaction of analytes in solutions and compare it with the state of the art (Legin et al., 1995, 1997, 1999a; Arrieta et al., 2004; Vlasov et al., 2005; Rudnitskaya and Legin, 2008; del Valle, 2012; Tahara and Toko, 2013). Technical improvements of the system have been illustrated in order to indicate possible measurement paths allowing better performances in terms of resolution and flexibility. The main additional characteristics are: the inclusion of a lock-in amplifier for the detection of signals when the signal to noise ratio is less than one; the presence of bias and sinusoidal input voltages for the reference electrode polarization; the possibility to divide the output signal in a high number of parts (till 1,000) in order to improve the resolution; the possibility to zoom in any point inside the response curve; the possibility to differentiate the output voltage in order to evidence details of the response; the use of light to change the sensitivity of the response (feature not utilized in this paper); the possibility to increase the input voltage dynamic range. An electronic chain comprising a spectrum analyzer has been included in the overall measurement system in order to evaluate the noise spectral density of the output amplifier with and without signals. Custom microfabricated electrodes were designed and realized to improve the performances of the sensing system.

A complete flow chart of the technological steps for the fabrication of miniaturized three electrode sensor has also been developed and reported.

The flexibility of the new ET system related to the utilization of these new miniaturized electrodes has been confirmed by showing the system performance/behavior/response in an application dealing with the detection of O_2_ and CO_2_ into a given solutions.

## Author contributions

GP: manuscript writing, electronic design, data interpretation; MS: manuscript writing, electronic design, device fabrication; LV: data analysis, data interpretation, manuscript revision; AZ: manuscript writing, device fabrication, data acquisition; AS and NK: device fabrication, data acquisition; IP: data analysis, manuscript revision; WQ: device fabrication; LS: electronic design, device fabrication, manuscript writing; AD: manuscript writing, electronic design, data interpretation.

### Conflict of interest statement

The authors declare that the research was conducted in the absence of any commercial or financial relationships that could be construed as a potential conflict of interest.
