# Supplementary material for: Advances in the Electronics for Cyclic Voltammetry: the Case of Gas Detection by Using Microfabricated Electrodes
Source: Front Chem. 2018 Aug 10;6:327. doi: 10.3389/fchem.2018.00327 (PMC6095978; doi:10.3389/fchem.2018.00327)
Supplement: Supplementary file 1 [file Data_Sheet_1.PDF]

Appendix

Micro-fabricated Electrodes

Specification on materials and processes

1.1 Oxide growth

- Furnaces Oxidation Time 16 min (initial thickness 25 Å; final thickness 3000 Å; temperature 1100°C; crystal orientation (1 0 0); environment wet).

1.2 Platinum steps

- Spin coating of the negative photoresist Nlof2020 (I step: 300 rpm; 10 sec;
  II step: 1500 rpm; 60 sec);
-
- Exposure of the photoresist with a negative mask *(Program: 2 (proximity), exposure time 5½ sec)*;
- Manual X-link bake (temperature 115°C, time 90 s);
- Development of the exposed photoresist *(Program Dev-lift-off);*
- Visual inspection;
- DUV bake;
- O2 Plasma flash;
- Evaporation of Pt on CHA (10 nm Tantalium and 300 nm of Platinum);
- Lift off procedure in an ultrasonic bath of NMP (N-metil-2-pirrolidone) solvent;
- Rinsing with DI water and drying.

1.3 Silver steps

- Treatment HMDS for 10 min in the manual HMDS unit;
- Manual spin coating of the negative photoresist Nlof2020 (I step: 300 rpm; 10 sec;
  II step: 1500 rpm; 60 sec);
- Pre soft bake (temperature: 95°C; time:60 sec);
- Exposure of the photoresist with a negative mask (*Prox, exposure time 6 sec*)
- Post soft bake (temperature: 115 °C; time: 90 sec);
- Development in MF322 (time: 110 sec);
- Visual inspection;
- Rinsing with DI water and drying;
- Hard bake (temperature: 100°C; time: 60 sec);

- Evaporation of Silver (10 nm Titanium and 300 nm of Silver);
- Lift off procedure in a ultrasonic bath of NMP (N-metil-2-pirrolidone) solvent;
- Rinsing with DI water and drying.

1.4 Gold steps

- Treatment HMDS for 10 min in the manual HMDS unit;
- Manual spin coating of the negative photoresist Nlof2020 (I step: 300 rpm; 10 sec;
  II step: 1500 rpm; 60 sec);
- Pre soft bake (temperature: 95°C; time: 60 sec);
- Post soft bake (temperature: 115 °C; time: 90 sec);
- Development in MF322 (time: 110 sec);
- Visual inspection;
- Rinsing with DI water and drying;
- Hard bake (temperature: 100°C; time: 60 sec);
- Evaporation of Silver (10 nm Titanium and 300 nm of Gold);
- Lift off procedure in an ultrasonic bath of NMP (N-metil-2-pirrolidone) solvent;
- Rinsing with DI water and drying.

1.5 Dicing

The wafers were diced and so for each wafer 16 electrodes were obtained.
